# The Influence of Transcranial Direct Current Stimulation on Shooting Performance in Elite Deaflympic Athletes: A Case Series

**DOI:** 10.3390/jfmk7020042

**Published:** 2022-05-25

**Authors:** Milan Pantovic, Drazenka Macak, Nebojsa Cokorilo, Sheniz Moonie, Zachary A. Riley, Dejan M. Madic, Brach Poston

**Affiliations:** 1 Department of Kinesiology and Nutrition Sciences, University of Nevada-Las Vegas, Las Vegas, NV 89154, USA; milan.pantovic@unlv.edu; 2 Faculty of Sport and Physical Education, University of Novi Sad, 21000 Novi Sad, Serbia; macak.md@yahoo.com (D.M.); dekimadic@gmail.com (D.M.M.); 3 Faculty of Sport, Union University-Nikola Tesla, 11000 Belgrade, Serbia; nebojsa.cokorilo@fzs.edu.rs; 4 Department of Epidemiology and Biostatistics, University of Nevada-Las Vegas, Las Vegas, NV 89154, USA; sheniz.moonie@unlv.edu; 5 Department of Kinesiology, Indiana University Purdue, Indianapolis, IN 46202, USA; zariley@iupui.edu

**Keywords:** motor skill, motor learning, transcranial direct current stimulation

## Abstract

Transcranial direct current stimulation (tDCS) has been shown to improve motor learning in numerous studies. However, only a few of these studies have been conducted on elite-level performers or in complex motor tasks that have been practiced extensively. The purpose was to determine the influence of tDCS applied to the dorsolateral prefrontal cortex (DLPFC) on motor learning over multiple days on 10-m air rifle shooting performance in elite Deaflympic athletes. Two male and two female elite Deaflympic athletes (World, European, and National medalists) participated in this case series. The study utilized a randomized, double-blind, SHAM-controlled, cross-over design. Anodal tDCS or SHAM stimulation was applied to the left DLPFC for 25 min with a current strength of 2 mA concurrent with three days of standard shooting practice sessions. Shooting performance was quantified as the points and the endpoint error. Separate 2 *Condition* (DLPFC-tDCS, SHAM) × 3 *Day* (1,2,3) within-subjects ANOVAs revealed no significant main effects or interactions for either points or endpoint error. These results indicate that DLPFC-tDCS applied over multiple days does not improve shooting performance in elite athletes. Different stimulation parameters or very long-term (weeks/months) application of tDCS may be needed to improve motor learning in elite athletes.

## 1. Introduction

Transcranial direct current stimulation (tDCS) is a non-invasive brain stimulation method that has been shown to improve motor skill and learning in numerous studies [1]. The vast majority of these studies have targeted the primary motor cortex (M1) with tDCS [1,2,3]. However, tDCS of other brain areas, such as the cerebellum [4,5,6,7,8,9], dorsolateral prefrontal cortex (DLPFC) [10,11,12,13,14,15], and supplementary motor area (SMA) [16,17], has also led to enhanced motor performance. The most common finding is that a 10 to 20-min tDCS application given simultaneously with motor practice improves motor skill by approximately 10% during and immediately after practice [1]. Furthermore, several studies involving either M1-tDCS [18,19] or cerebellar tDCS [5] applied over three to five consecutive days have shown that the total amount of motor learning experienced by subjects can be increased by 20–30% compared to SHAM stimulation, although the number of multi-day studies is small in comparison to single day studies.

Despite the aforementioned promising findings, these studies have had several interrelated limitations that make it difficult to determine the degree of viability of tDCS as an adjunct intervention to improve motor skill and learning in real-world applications, such as in sport, military, and workplace settings. First, the motor tasks practiced were relatively simple and usually involved either one to four digits of the hand, a single joint or limb, isometric contractions, or some combination of these conditions. Second, the tasks were often novel laboratory tasks that the participants had likely never done before in everyday life. Third, the participants were usually novice performers of the motor task. A very small number of tDCS studies in novices have at least examined more complex multi-joint movements using a rather wide variety of stimulation parameters but have reported mixed findings [4,14,20,21,22,23]. Thus, complex multi-joint tasks that have been extensively practiced have rarely been investigated in tDCS studies involving motor learning, especially in elite performers or athletes.

The **purpose** was to determine the effects of DLPFC-tDCS on motor learning over multiple days on 10-m air rifle shooting performance in elite Deaflympic athletes. This was accomplished by having participants complete a set of practice sessions in a DLPFC-tDCS condition and a SHAM condition in a cross-over design with a week washout period. Based on previous single session DLPFC-tDCS studies that involved relatively simple motor tasks in healthy young adults [10,11,15] and studies in relatively novice shooters [12,13], it was hypothesized that DLPFC-tDCS would enhance shooting performance to a greater degree compared to practice alone (SHAM stimulation). Specifically, it was expected that shooting performance would progressively improve over the three days of DLPFC-tDCS application, whereas shooting performance would remain relatively constant over the course of the three days of SHAM stimulation. It was also predicted that shooting performance would remain higher for at least one practice day following the end of the stimulation sessions for the DLPFC-tDCS condition compared to the SHAM condition.

The left DLPFC was targeted with anodal tDCS as opposed to other stimulation montages (e.g., cathodal tDCS of left or right DLPFC) primarily because the greatest number of both motor skill [10,11,15] and gross motor [24,25,26] studies had used this set of parameters. In addition, DLPFC-tDCS was given during task practice as opposed to before or during, as previous studies have achieved the best results when tDCS is applied concurrently with motor practice [5,18,19]. Finally, air rifle shooting was chosen as the motor task because it is a real-world, complex task that involves visuo-motor integration, coordination of both limbs, and strict concurrent postural muscle control.

## 2. Materials and Methods

### 2.1. Participants

A total of 4 elite Deaflympic 10-m air rifle athletes (2 female, 2 men) volunteered to participate in the study and provided informed written consent. All subjects were right-handed and right-handed shooters. The study was approved by the ethical committee of the institutional ethics committee from the Faculty of Sport and Physical Education, University of Novi Sad, Serbia (protocol number: 1/2021), and was conducted in accordance with the Declaration of Helsinki. All of the participants had undergone extensive multi-year training and had substantial competitive shooting experience, including being medalists at the National, European, and World Championships levels (see below).

- ID 1—37-year-old female with 14 years of training experience. She won the gold medal in the 10-m air rifle at the 2014 European Deaf Shooting Championships.
- ID 2—42-year-old female with 18 years of training experience. She won bronze medals at the 2009 and 2013–Deaflympics, 2015—European Deaf Shooting Championships (2× bronze medals and 2× silver medals), a silver medal at the 2016 World Deaf Shooting Championships, and a silver medal in the mixed 10-m air rifle at the 2019 European Deaf Shooting Championships.
- ID 3—26-year-old man with 12 years of training experience. He won the silver medal in the 10-m air rifle in the 2019 International Competitions “Istvan Poljanac”.
- ID 4—23-year-old man with 12 years of training experience. He won the silver medal in the mixed 10-m air rifle at the 2019 European Deaf Shooting Championships.

### 2.2. Experimental Design

**Figure 1:**
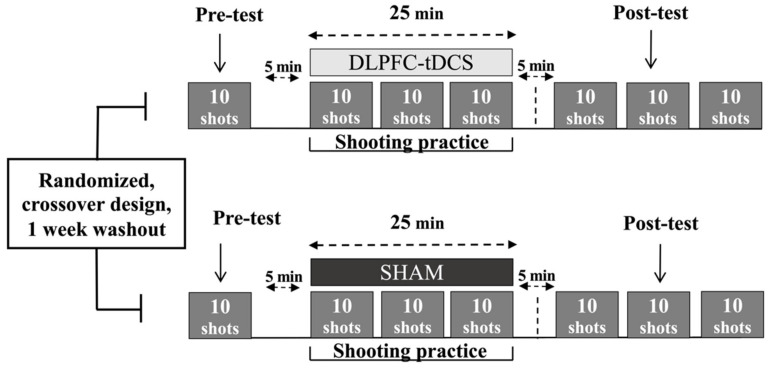
Experimental design. A schematic representation of the experimental protocol for a single practice session for each of the two conditions (DLPFC-tDCS, SHAM) is depicted for illustrative purposes, although an identical protocol was performed in each condition for 3 consecutive days. A week washout period between the two 3-Day series of practice sessions was implemented after which the participants crossed over to the opposite condition.

The study was a case series that utilized a randomized, double-blind, SHAM-controlled, within-subjects, cross-over design. A schematic of the overall experimental design and schedule is depicted in Figure 1. The 4 participants each took part in a total of 6 practice sessions consisting of 3 consecutive days of DLPFC-tDCS and 3 consecutive days of SHAM stimulation with a week washout period between the two series of practice sessions. All practice sessions were performed at the same training facility in which the athletes performed their normal training regiment. The order of the experimental conditions was randomized. The randomization sequence was generated for the 4 participants by a computer (http://www.randomization.com/ accessed on 1 March 2021) using random balanced permutations. Thus, 2 participants performed the DLPFC-tDCS condition first and the SHAM condition second, whereas the other 2 participants performed the series of practice sessions in the opposite order. An investigator who did not participate in data collection or data analysis programmed the stimulator in each session. Therefore, the investigators who collected and analyzed data were blinded to the experimental conditions.

### 2.3. DLPFC-tDCS

A Caputron tDCS Stimulator was placed in a small, tight-fitting backpack so that shooting performance was not restricted. The location of DLPFC was determined using the methodology of the Beam F3 system [27]. Briefly, the investigators took head measurements with a measuring tape: (1) Tragus-tragus, (2) nasion-inion, and (3) the head circumference. The values were entered in the free software program www.clinicalresearcher.org. The program then calculated the x, y coordinates for the F3 location (according to the international 10–20 system) of DLPFC for each participant. Anodal DLPFC-tDCS was delivered using previously determined effective parameters for improving fine and gross motor performance (duration 25 min; current 2 mA; anode over left DLPFC; cathode over the contralateral supra-orbital region) [10,11,15,24,25,26]. Thus, the stimulation was applied to the DLPFC of the dominant hemisphere-arm system as all subjects were right-handed. The current was delivered through two rubber electrodes (5 × 5 cm) enclosed in saline-soaked sponges that were held in place with a pair of rubber straps. For SHAM, the current was ramped up and down over 30 s according to standard procedures for SHAM stimulation in tDCS studies [28]. The left DLPFC was targeted with anodal tDCS for several interrelated reasons: (1) Several studies have shown that left DLPFC-tDCS can improve fine motor [10,11,15] and gross motor performance [24,25,26], (2) methodologies have been determined using simple measuring equipment for the accurate placement of the tDCS electrodes for DLPFC [27] without the need for expensive equipment that was unavailable at the athlete’s training facility. For instance, the need to use transcranial magnetic stimulation (TMS) to find the motor hot spot for M1-tDCS, and (3) the left DLPFC has ipsilateral connections to several brain regions, including premotor cortex, basal ganglia, cerebellum, and SMA [29], which likely partially explains its role in motor planning and motor learning processes [29,30] and its indirect influence on M1 [29].

### 2.4. Practice Sessions

The air rifle shooting task was executed by Deaflympic event rules (International Shooting Sport Federation Rules and Regulations) in which the athletes try to hit a stationary electronic target from a distance of 10 m. The diameter of the center of the 10-ring target was 0.5 mm. Each practice session consisted of a pre-test block, 3 practice blocks, and 3 post-test blocks (Figure 1). First, participants performed the pre-test block (10 trials) without stimulation but with the inert tDCS montage placed on the head to mimic the same conditions as the practice/stimulation blocks. Second, after a five-minute break, subjects received DLPFC-tDCS or SHAM stimulation while performing 3 blocks (practice blocks) of shooting trials over a maximum period of 25 min in the same manner in which they train/compete. Next, another five-minute break was undertaken. Third, participants performed an additional 3 blocks (post-test blocks). These blocks were also performed without stimulation but with the now inert DLPFC-tDCS montage still on the head. In the 25 min stimulation time periods (second step above), the stimulator was first allowed to run for 3 min, the shooting blocks took approximately 5 min (1 shot every 30 s), and there was a 2 min rest period between the shooting blocks. This assured that all the trials could be performed with a few minutes to spare until the 25 min stimulation time elapsed. In all trials, the participants used visual feedback of the projectile endpoint relative to the electronic target center after each trial using the SIUS SA951 (SIUS AG, Switzerland) software and hardware system to facilitate the goal of minimizing error distance on subsequent attempts.

Rifle shooting was selected as the motor task for the following reasons: (1) Rifle shooting is a real-world, difficult motor task that involves visuo-motor integration, coordination of both limbs, and appropriate postural muscle activation, (2) the availability and willingness of this group of Deaflympic athletes and their coaches to participate in the study during their normal training routine, and (3) pistol shooting performance was able to be enhanced in previous DLPFC-tDCS studies in novices, although somewhat different stimulation parameters were used [12,13].

### 2.5. Data Analysis

The dependent variables were the points and the endpoint error. The points were calculated for each trial block according to the scoring system used in training and competition by the athletes. Accordingly, points were awarded on a 0 to 10.9-point scale based on the distance of the endpoint of each shot from the center of the target for each trial. Thus, the highest possible score in a block of trials was 109 total points (10 trials × 10.9 points). The average points scored in the 10 shooting trials in each block were taken as the points scored and used for analysis. Endpoint error was calculated according to previous studies [4,21,31]. Briefly, the shortest distance between the final endpoint *x*, *y* coordinates of each shot relative to the *x*, *y* coordinates of the center of the target was calculated for each block using the Pythagorean Theorem. For an in-depth description of the steps involved in quantifying endpoint error, see Poston et al. (2013) [32]. The average endpoint error of the 10 shooting trials in each block was taken as the endpoint error value and used for analysis.

### 2.6. Statistical Analysis

The dependent variables of points and endpoint error were analyzed with separate 2 *Condition* (DLPFC-tDCS, SHAM) × 3 *Day* (1,2,3) within-subjects ANOVAs.

## 3. Results

### 3.1. Group Level Observations

**Figure 2:**
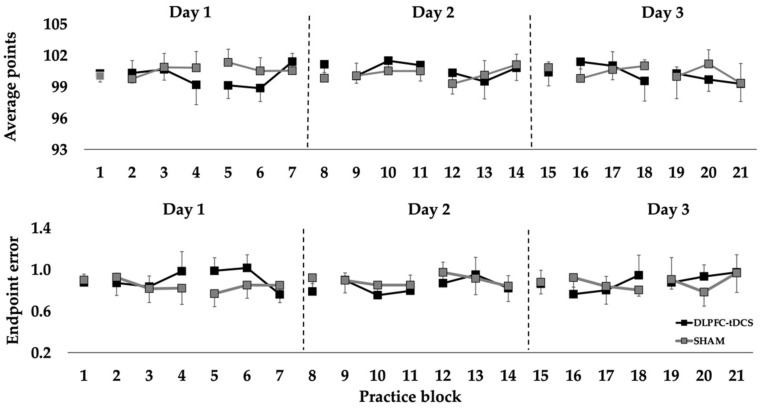
Points and endpoint error in the rifle shooting task in the pre-test, practice, and post-test blocks over 3 consecutive days for the DLPFC-tDCS and SHAM conditions. Each point represents the average of a block of 10 rifle shooting trials.

For average points, the *Condition* main effect (*p* = 0.333), *Day* main effect (*p* = 0.478), and *Condition* × *Day* interaction (*p* = 0.338) were all non-statistically significant. Similarly, the *Condition* main effect (*p* = 0.814), *Day* main effect (*p* = 0.841), and *Condition* × *Day* interaction (*p* = 0.219) were all non-statistically significant for endpoint error (Figure 2).

### 3.2. Individual Data

Due to the limitations of using statistical tests yielding *p* values for case series data, the points and endpoint error (daily averages of all trial blocks) of each participant for each practice day are presented for the DLPFC-tDCS and SHAM conditions in Table 1. In Table 2, the total points and total endpoint error (3-Day grand averages) for the DLPFC-tDCS and SHAM conditions are presented.

- ID 1 scored more points in the DLPFC-tDCS condition on Days 2 and 3. However, the 3-Day total points score was slightly higher in the SHAM condition (708 vs. 707.4 points). Similarly, the total endpoint error was lower during the DLPFC-tDCS condition on Days 2 and 3. However, the total endpoint error was lower in the SHAM condition (5.5 vs. 5.6 mm).
- ID 2 scored more points in the SHAM condition on Days 1 and 3. Moreover, the 3-Day total points score was slightly higher in the SHAM condition (705.8 vs. 704.1 points). Similarly, the total endpoint error was lower during the SHAM condition on Days 1 and 3. However, the total endpoint error was lower in the SHAM condition (5.6 vs. 5.9 mm).
- ID 3 scored more points in the SHAM condition on Days 2 and 3. Moreover, the 3-Day total points score was slightly higher in the SHAM condition (682.9 vs. 679.3 points). Similarly, the total endpoint error was lower during the SHAM condition on Days 2 and 3. However, the total endpoint error was lower in the SHAM condition (8 vs. 8.1 mm).
- ID 4 scored more points in the DLPFC-tDCS condition on Days 2 and 3. In addition, the 3-Day total points score was slightly higher in the DLPFC-tDCS condition (716.6 vs. 713.6 points). Similarly, the total endpoint error was lower during the DLPFC-tDCS condition on Days 2 and 3. However, the total endpoint error was lower in the DLPFC-tDCS condition (4.6 vs. 5 mm).

## 4. Discussion

The purpose was to determine the effects of DLPFC-tDCS on motor learning over multiple days on 10-m air rifle shooting performance in elite Deaflympic athletes. There were three main findings. First, DLPFC-tDCS applied concurrently with practice over three practice sessions did not improve total points or endpoint error relative to SHAM stimulation. Second, total points and endpoint error were similar in the DLPFC-tDCS condition and the SHAM condition in the post-test blocks performed after stimulation on each of the three days. Third, shooting performance remained relatively constant across all practice days and practice blocks in both stimulation conditions and near the highest levels attained by these athletes in training and competition. Taken together, the findings indicate that DLPFC-tDCS applied concurrently with practice for three consecutive days does not improve shooting performance in elite athletes beyond performance ceiling levels reached through extensive practice using traditional training approaches.

### 4.1. Influence of DLPFC-tDCS on Motor Skill and Learning in Rifle Shooting

The majority of studies that have applied tDCS to M1 or the cerebellum in novices have observed acute enhancements in motor skill when measured during and immediately after stimulation [1,4]. Although far fewer tDCS studies have targeted DLPFC, several have demonstrated acute improvements in various fine motor skills [10,11,15]. Based on these studies, it was originally hypothesized that DLPFC-tDCS applied concurrently with the practice blocks would improve shooting accuracy compared with the SHAM condition. In addition, it was predicted that shooting accuracy would be greater in the DLPFC-tDCS condition in the post-test blocks, based on previous studies that had shown tDCS induced skill enhancements for about 30–45 min after cessation of stimulation. Contrary to this hypothesis, shooting performance as quantified by both points and endpoint error was almost identical between the DLPFC-tDCS and SHAM conditions in both the practice/stimulation blocks and post-test blocks.

These findings differ from several previous single session DLPFC-tDCS studies that also targeted left DLPFC with anodal tDCS in fine motor tasks. For instance, Grospretre et al. (2021) reported that left DLPFC-tDCS enhanced performance in a Fitt’s type pointing task performed with the right hand and arm [10], whereas Hsu et al. (2015) observed improved multi-tasking performance in a 3-D video game involving visuomotor tracking [11]. In addition, Jin et al. (2019) [15] found that DLPFC-tDCS augmented force control in a bimanual isometric force production task. The current findings are also in contrast to a series of studies that involved anodal left DLPFC-tDCS and gross motor performance. Specifically, fatiguability, as measured either by the time to task failure or the number of repetitions performed, was enhanced in a lower body cycle ergometer task [25], bicep curls [24], and the bench press exercise [26]. However, it is difficult to determine how applicable these results are to the current results as the neuromuscular mechanisms underlying fine motor skills performed in a non-fatigued state are much different to gross motor skills done to volitional fatigue.

The present findings also differ from motor skill studies that have applied tDCS to DLPFC but using alternative stimulation parameters. For example, an acute application of cathodal tDCS of the left DLPFC improved golf putting performance in novice golfers [14]. Similarly, a one-time application of anodal tDCS applied to the right DLPFC augmented pistol shooting performance in unskilled shooters [13]. In another single-session study involving pistol shooting [12], a novel electrode montage was employed where the anode was placed over the right cerebellum and the cathode over the left DLPFC, which would theoretically inhibit the left DLPFC. The results indicated that this arrangement improved shooting accuracy in club-level shooters compared to SHAM stimulation. Nonetheless, it is difficult to reconcile the results of this study with the current findings as it is likely that the improvement in performance could have been at least partially mediated through the cerebellar stimulation and the shooters were apparently not national or international level performers.

Despite the aforementioned disparate findings, the present results are in agreement with Vancleef et al. (2016), who reported that anodal left DLPFC-tDCS applied for four consecutive days failed to improve performance in a complex, bimanual visuomotor tracking task that was novel to the participants prior to the study [20]. Similarly, left DLPFC-tDCS did not enhance manual dexterity task performance (grooved pegboard test), albeit in older adults [33]. Finally, professional piano players underwent a single session of M1-tDCS in association with execution of piano sequences [23] in one of the only tDCS studies that have investigated fine motor skill acquisition in expert performers. The findings indicated that M1-tDCS did not improve the performance of the motor task compared to SHAM. However, the participants of that study were individuals with focal hand dystonia, which may have precluded the ability of tDCS to improve performance in a single session. Collectively, those results and the current findings suggest that DLPFC-tDCS, and perhaps tDCS of other brain areas, may not be able to enhance motor learning in complex or extensively practiced tasks, especially in elite performers.

### 4.2. Possible Factors Responsible for Inability of DLPFC-tDCS to Improve Shooting Performance

The failure of DLPFC-tDCS to enhance rifle shooting performance in the current study is not consistent with the majority of the tDCS literature but could be due to several interrelated factors. First, the most obvious explanation is that DLPFC-tDCS is simply not able to improve motor performance in elite performers due to ceiling effects from investigating a motor task practiced extensively over many years. Accordingly, the handful of tDCS studies that have investigated complex motor tasks either involved tasks completely novel to the participants or ones with which they had experience in the past, but were not performing regularly at the time of the study [4]. Second, the number of stimulation days may not have been sufficient to improve shooting performance. Although three to five days of tDCS has elicited large performance increases in simple motor tasks in young adults [5,18,19], it could be argued that as many as 9–40 days of tDCS could be needed to see an effect. Many studies in patient populations have utilized this range of stimulation sessions but have reported mixed results [34,35,36]. Third, interindividual differences in the responsiveness to DLPFC-tDCS could be at least partially responsible for the lack of observable performance enhancements. Accumulating evidence suggests that variations in several anatomical, biological, and physiological features (e.g., skull and cerebrospinal fluid thickness as well as neuronal orientation and neurotransmitter levels) could influence tDCS outcomes [37,38,39]. These factors could have collectively influenced the total amount and distribution of current reaching the brain area of interest. Thus, it is possible that some of the participants in the current study could have been non-responders to DLPFC-tDCS. However, almost all studies on this topic made this classification based solely on acute TMS cortical excitability measures in response to tDCS and did not measure motor performance at all [40,41]. Furthermore, an extensive study demonstrated that these same cortical excitability measures actually showed no relationship to the amount of motor learning achieved due to tDCS [42]. In contrast, several issues that are commonly cited in tDCS studies that fail to elicit performance improvements are not applicable to the current study. For instance, the use of a within-subject cross-over design eliminated the major issue of possible genetic differences [38,39,43] that arise in between-subject designs involving separate subject groups. The issues of time of day and possible neurodegeneration of brain areas in advancing age [37] or motor disorders also do not apply as all experiments were done at the same time of day, and the participants were all healthy, free of neurological disorders, and young or middle aged.

### 4.3. Limitations

The study had several limitations that should be acknowledged. The major limitation was that this was a case series with very low sample size, as can be expected with finding elite-level achievers in any cognitive or physical domain. Accordingly, this could have constrained the ability to observe a significant influence of DLPFC-tDCS on rifle shooting performance in this population. Nonetheless, there was little, if any, indication that DLPFC-tDCS induced any trend for enhanced performance during or after stimulation in any subject. Another limitation was that only one potential brain area was targeted with only one of several possible sets of stimulation parameters for DLPFC-tDCS. A final limitation was the relatively small number of stimulation sessions. Although the current study is one of only a handful of tDCS studies that have involved three or more sessions in healthy young adults, it could be argued that two to six weeks of tDCS application may be needed to significantly improve motor learning in well-practiced tasks in non-novice performers.

## 5. Conclusions

In summary, DLPFC-tDCS applied for three consecutive days simultaneously with typical practice sessions did not improve rifle shooting performance in elite Deaflympic athletes. The findings are in contrast to single session DLPFC-tDCS [10,11,15] and M1-tDCS studies performed over several days [18,19] that have found significantly enhanced motor learning outcomes. However, the findings are consistent with a previous study involving multi-day DLPFC-tDCS in a complex motor task in young adults [20] and an M1-tDCS study utilizing an extensively practiced task in expert performers [23]. Subsequent studies may need to use different parameters of DLPFC-tDCS or target different brain regions (M1, cerebellum) in relatively long-term (weeks or months) intervention periods to be able to observe noticeable performance enhancements in elite populations. Finally, future work needs to examine a large sample of elite performers, although it is extremely challenging to recruit a large sample of elite athletes for long-term trials that could potentially interfere with their normal training regiments.

## Figures and Tables

**Table 1:** Points and endpoint error for each practice day in the DLPFC-tDCS and SHAM conditions.

	Points						Endpoint Error (mm)					
	tDCS			SHAM			tDCS			SHAM		
Subject	Day 1	Day 2	Day 3	Day 1	Day 2	Day 3	Day 1	Day 2	Day 3	Day 1	Day 2	Day 3
**ID 1**	699.7	711.7	710.8	710.2	703.2	710.6	6.33	5.13	5.23	5.28	5.98	5.24
**ID 2**	701.6	705.7	705.1	707.4	704.4	705.6	6.14	5.73	5.79	5.55	5.86	5.37
**ID 3**	685	680.6	672.2	683.4	684.4	681	7.8	8.05	8.55	7.96	7.9	8.28
**ID 4**	712.7	719.2	717.9	714.1	713.3	713.5	5.03	4.38	4.51	4.88	4.97	5.01
**Average**	699.8	704.3	701.5	703.8	701.3	702.7	6.3	5.8	6.0	5.9	6.2	6.0

**Table 2:** 3-Day total points and total endpoint error along with the SD, CV, and confidence intervals for DLPFC-tDCS and SHAM conditions.

		Total Points					Total Endpoint Error (mm)				
					Confidence Interval					Confidence Interval	
Subject	Condition	Mean	SD	CV	LL 95%	HL 95%	Mean	SD	CV	LL 95%	HL 95%
**ID 1**	**tDCS**	707.4	6.7	0.9	690.8	724.0	5.6	0.7	12	3.9	7.2
	**SHAM**	708.0	4.2	0.6	697.7	718.3	5.5	0.4	7.6	4.5	6.5
**ID 2**	**tDCS**	704.1	2.2	0.3	698.6	709.6	5.9	0.2	3.8	5.3	6.4
	**SHAM**	705.8	1.5	0.2	702.0	709.6	5.6	0.2	4.4	5	6.2
**ID 3**	**tDCS**	679.3	6.5	1	663.1	695.4	8.1	0.4	4.7	7.2	9.1
	**SHAM**	682.9	1.7	0.3	678.6	687.3	8	0.2	6.8	7.5	8.6
**ID 4**	**tDCS**	716.6	3.4	0.5	708.1	725.1	4.6	0.3	7.4	3.8	5.5
	**SHAM**	713.6	0.4	0.1	712.6	714.7	5	0.1	1.3	4.8	5.1

## Data Availability

The data presented in the study are available on request from the corresponding author.

## References

[1] Buch E.R., Santarnecchi E., Antal A., Born J., Celnik P.A., Classen J., Gerloff C., Hallett M., Hummel F.C., Nitsche M.A. (2017). Effects of tDCS on motor learning and memory formation: A consensus and critical position paper. Clin. Neurophysiol..

[2] Nitsche M.A., Paulus W. (2011). Transcranial Direct Current Stimulation—Update 2011. Restor. Neurol. Neurosci..

[3] Stagg C.J., Nitsche M.A. (2011). Physiological Basis of Transcranial Direct Current Stimulation. Neuroscientist.

[4] Jackson A.K., de Albuquerque L.L., Pantovic M., Fischer K.M., Guadagnoli M.A., Riley Z.A., Poston B. (2019). Cerebellar Transcranial Direct Current Stimulation Enhances Motor Learning in a Complex Overhand Throwing Task. Cerebellum.

[5] Cantarero G., Spampinato D., Reis J., Ajagbe L., Thompson T., Kulkarni K., Celnik P. (2015). Cerebellar Direct Current Stimulation Enhances on-Line Motor Skill Acquisition through an Effect on Accuracy. J. Neurosci..

[6] Celnik P. (2015). Understanding and Modulating Motor Learning with Cerebellar Stimulation. Cerebellum.

[7] Grimaldi G., Argyropoulos G., Boehringer A., Celnik P., Edwards M.J., Ferrucci R., Galea J.M., Groiss S.J., Hiraoka K., Kassavetis P. (2014). Non-invasive Cerebellar Stimulation—A Consensus Paper. Cerebellum.

[8] Oldrati V., Schutter D.J.L.G. (2018). Targeting the Human Cerebellum with Transcranial Direct Current Stimulation to Modulate Behavior: A Meta-Analysis. Cerebellum.

[9] Van Dun K., Bodranghien F.C.A.A., Mariën P., Manto M.U. (2016). tDCS of the Cerebellum: Where Do We Stand in 2016? Technical Issues and Critical Review of the Literature. Front. Hum. Neurosci..

[10] Grospretre S., Grandperrin Y., Nicolier M., Gimenez P., Vidal C., Tio G., Haffen E., Bennabi D. (2021). Effect of Transcranial Direct Current Stimulation on the Psychomotor, Cognitive, and Motor Performances of Power Athletes. Sci. Rep..

[11] Hsu W.-Y., Zanto T.P., Anguera J.A., Lin Y.-Y., Gazzaley A. (2015). Delayed enhancement of multitasking performance: Effects of anodal transcranial direct current stimulation on the prefrontal cortex. Cortex.

[12] Kamali A.-M., Nami M., Yahyavi S.-S., Saadi Z.K., Mohammadi A. (2019). Transcranial Direct Current Stimulation to Assist Experienced Pistol Shooters in Gaining Even-Better Performance Scores. Cerebellum.

[13] Rocha K., Marinho V., Magalhães F., Carvalho V., Fernandes T., Ayres M., Crespo E., Velasques B., Ribeiro P., Cagy M. (2020). Unskilled shooters improve both accuracy and grouping shot having as reference skilled shooters cortical area: An EEG and tDCS study. Physiol. Behav..

[14] Zhu F.F., Yeung A.Y., Poolton J.M., Lee T.M., Leung G.K., Masters R.S. (2015). Cathodal Transcranial Direct Current Stimulation over Left Dorsolateral Prefrontal Cortex Area Promotes Implicit Motor Learning in a Golf Putting Task. Brain Stimul..

[15] Jin Y., Lee J., Kim S., Yoon B. (2019). Noninvasive Brain Stimulation over M1 and Dlpfc Cortex Enhances the Learning of Bimanual Isometric Force Control. Hum. Mov. Sci..

[16] Hupfeld K., Ketcham C.J., Schneider H.D. (2017). Transcranial direct current stimulation (tDCS) to the supplementary motor area (SMA) influences performance on motor tasks. Exp. Brain Res..

[17] Vollmann H., Conde V., Sewerin S., Taubert M., Sehm B., Witte O.W., Villringer A., Ragert P. (2013). Anodal transcranial direct current stimulation (tDCS) over supplementary motor area (SMA) but not pre-SMA promotes short-term visuomotor learning. Brain Stimul..

[18] Reis J., Fischer J.T., Prichard G., Weiller C., Cohen L.G., Fritsch B. (2015). Time-but Not Sleep-Dependent Consolidation of Tdcs-Enhanced Visuomotor Skills. Cereb. Cortex.

[19] Reis J., Schambra H.M., Cohen L.G., Buch E.R., Fritsch B., Zarahn E., Celnik P.A., Krakauer J.W. (2009). Noninvasive cortical stimulation enhances motor skill acquisition over multiple days through an effect on consolidation. Proc. Natl. Acad. Sci. USA.

[20] Vancleef K., Meesen R., Swinnen S.P., Fujiyama H. (2016). tDCS over left M1 or DLPFC does not improve learning of a bimanual coordination task. Sci. Rep..

[21] Albuquerque L.L., Fischer K.M., Pauls A.L., Pantovic M., Guadagnoli M.A., Riley Z.A., Poston B. (2019). An acute application of transcranial random noise stimulation does not enhance motor skill acquisition or retention in a golf putting task. Hum. Mov. Sci..

[22] Meek A.W., Greenwell D., Poston B., Riley Z.A. (2021). Anodal tDCS accelerates on-line learning of dart throwing. Neurosci. Lett..

[23] Buttkus F., Baur V., Jabusch H.C., de la Cruz Gomez-Pellin M., Paulus W., Nitsche M.A., Altenmuller E. (2011). Single-Session Tdcs-Supported Retraining Does Not Improve Fine Motor Control in Musician’s Dystonia. Restor. Neurol. Neurosci..

[24] Lattari E., Andrade M.L., Filho A.S., Moura A.M., Neto G.M., Silva J.G., Rocha N.B., Yuan T.F., Arias-Carrion O., Machado S. (2016). Can Transcranial Direct Current Stimulation Improve the Resistance Strength and Decrease the Rating Perceived Scale in Recreational Weight-Training Experience?. J. Strength Cond. Res..

[25] Lattari E., de Oliveira B.S., Oliveira B.R.R., de Mello Pedreiro R.C. (2018). Effects of transcranial direct current stimulation on time limit and ratings of perceived exertion in physically active women. Neurosci. Lett..

[26] Alix-Fages C., García-Ramos A., Calderón-Nadal G., Colomer-Poveda D., Romero-Arenas S., Fernández-Del-Olmo M., Márquez G. (2020). Anodal transcranial direct current stimulation enhances strength training volume but not the force–velocity profile. Eur. J. Appl. Physiol..

[27] Beam W., Borckardt J.J., Reeves S.T., George M.S. (2009). An efficient and accurate new method for locating the F3 position for prefrontal TMS applications. Brain Stimul..

[28] Nitsche M.A., Cohen L.G., Wassermann E.M., Priori A., Lang N., Antal A., Paulus W., Hummel F., Boggio P.S., Fregni F. (2008). Transcranial direct current stimulation: State of the art 2008. Brain Stimul..

[29] Wang Y., Cao N., Lin Y., Chen R., Zhang J. (2020). Hemispheric Differences in Functional Interactions Between the Dorsal Lateral Prefrontal Cortex and Ipsilateral Motor Cortex. Front. Hum. Neurosci..

[30] Kantak S.S., Sullivan K.J., E Fisher B., Knowlton B.J., Winstein C.J. (2010). Neural substrates of motor memory consolidation depend on practice structure. Nat. Neurosci..

[31] Lima de Albuquerque L., Pantovic M., Clingo M., Fischer K., Jalene S., Landers M., Mari Z., Poston B. (2020). An Acute Application of Cerebellar Transcranial Direct Current Stimulation Does Not Improve Motor Performance in Parkinson’s Disease. Brain Sci..

[32] Poston B., Van Gemmert A.W., Sharma S., Chakrabarti S., Zavaremi S.H., Stelmach G. (2013). Movement trajectory smoothness is not associated with the endpoint accuracy of rapid multi-joint arm movements in young and older adults. Acta Psychol..

[33] Ljubisavljevic M.R., Oommen J., Filipovic S., Bjekic J., Szolics M., Nagelkerke N. (2019). Effects of tDCS of Dorsolateral Prefrontal Cortex on Dual-Task Performance Involving Manual Dexterity and Cognitive Task in Healthy Older Adults. Front. Aging Neurosci..

[34] De Albuquerque L.L., Pantovic M., Clingo M.G., Fischer K.M., Jalene S., Landers M.R., Mari Z., Poston B. (2021). Long-Term Application of Cerebellar Transcranial Direct Current Stimulation Does Not Improve Motor Learning in Parkinson’s Disease. Cerebellum.

[35] Valentino F., Cosentino G., Brighina F., Pozzi N.G., Sandrini G., Fierro B., Savettieri G., D’Amelio M., Pacchetti C. (2014). Transcranial direct current stimulation for treatment of freezing of gait: A cross-over study. Mov. Disord..

[36] Shibata T., Urata A., Kawahara K., Furuya K., Ishikuro K., Hattori N., Kuroda S. (2020). Therapeutic Effects of Diagonal-Transcranial Direct Current Stimulation on Functional Recovery in Acute Stroke: A Pilot Study. J. Stroke Cerebrovasc. Dis..

[37] Miterko L.N., Baker K.B., Beckinghausen J., Bradnam L.V., Cheng M.Y., Cooperrider J., DeLong M.R., Gornati S.V., Hallett M., Heck D.H. (2019). Consensus Paper: Experimental Neurostimulation of the Cerebellum. Cerebellum.

[38] Pellegrini M., Zoghi M., Jaberzadeh S. (2018). Biological and anatomical factors influencing interindividual variability to noninvasive brain stimulation of the primary motor cortex: A systematic review and meta-analysis. Rev. Neurosci..

[39] Li L., Uehara K., Hanakawa T. (2015). The contribution of interindividual factors to variability of response in transcranial direct current stimulation studies. Front. Cell. Neurosci..

[40] Labruna L., Jamil A., Fresnoza S., Batsikadze G., Kuo M.-F., Vanderschelden B., Ivry R.B., Nitsche M.A. (2016). Efficacy of Anodal Transcranial Direct Current Stimulation is Related to Sensitivity to Transcranial Magnetic Stimulation. Brain Stimul..

[41] Wiethoff S., Hamada M., Rothwell J.C. (2014). Variability in Response to Transcranial Direct Current Stimulation of the Motor Cortex. Brain Stimul..

[42] López-Alonso V., Cheeran B., Fernández-Del-Olmo M. (2015). Relationship Between Non-invasive Brain Stimulation-induced Plasticity and Capacity for Motor Learning. Brain Stimul..

[43] MacInnis M.J., McGlory C., Gibala M.J., Phillips S.M. (2017). Investigating human skeletal muscle physiology with unilateral exercise models: When one limb is more powerful than two. Appl. Physiol. Nutr. Metab..

